# Triglyceride to HDL Cholesterol Ratio for the Identification of MASLD in Obesity: A Liver Biopsy-Based Case-Control Study

**DOI:** 10.3390/nu16091310

**Published:** 2024-04-27

**Authors:** José Ignacio Martínez-Montoro, María Antonia Martínez-Sánchez, Andrés Balaguer-Román, Virginia Esperanza Fernández-Ruiz, José Emilio Hernández-Barceló, Mercedes Ferrer-Gómez, María Dolores Frutos, María Ángeles Núñez-Sánchez, José Carlos Fernández-García, Bruno Ramos-Molina

**Affiliations:** 1 Department of Endocrinology and Nutrition, Virgen de la Victoria University Hospital, Instituto de Investigación Biomédica de Málaga y Plataforma en Nanomedicina-IBIMA Plataforma BIONAND, Faculty of Medicine, University of Malaga, 29010 Malaga, Spain; joseimartinezmontoro@gmail.com; 2 Obesity, Diabetes and Metabolism Laboratory, Biomedical Research Institute of Murcia (IMIB), 30120 Murcia, Spain; mariaantonia.martinez1@um.es (M.A.M.-S.); a.balaguerroman@gmail.com (A.B.-R.); virginiaesperanza.fernandez@um.es (V.E.F.-R.); mercedesferrer@um.es (M.F.-G.);; 3 Department of General and Digestive System Surgery, Virgen de la Arrixaca University Hospital, 30120 Murcia, Spain; mfb25s@gmail.com; 4 Department of Endocrinology and Nutrition, Virgen de la Arrixaca University Hospital, 30120 Murcia, Spain; 5 Department of Pathology, Virgen de la Arrixaca University Hospital, 30120 Murcia, Spain; joseemilio99@yahoo.es; 6 Department of Endocrinology and Nutrition, Regional University Hospital of Malaga, Instituto de Investigación Biomédica de Málaga y Plataforma en Nanomedicina-IBIMA Plataforma BIONAND, Faculty of Medicine, University of Malaga, 29010 Malaga, Spain

**Keywords:** metabolic dysfunction-associated steatotic liver disease, obesity, TG/HDL-C ratio, metabolic dysfunction-associated steatohepatitis, liver biopsy

## Abstract

Associations between dyslipidemia and metabolic dysfunction-associated steatotic liver disease (MASLD) have been reported. Previous studies have shown that the triglyceride to high-density lipoprotein cholesterol (TG/HDL-C) ratio may be a surrogate marker of MASLD, assessed by liver ultrasound. However, no studies have evaluated the utility of this ratio according to biopsy-proven MASLD and its stages. Therefore, our aim was to evaluate if the TG/HDL-C ratio allows for the identification of biopsy-proven MASLD in patients with obesity. We conducted a case-control study in 153 patients with obesity who underwent metabolic surgery and had a concomitant liver biopsy. Fifty-three patients were classified as no MASLD, 45 patients as metabolic dysfunction-associated steatotic liver—MASL, and 55 patients as metabolic dysfunction-associated steatohepatitis—MASH. A receiver operating characteristic (ROC) analysis was performed to assess the accuracy of the TG/HDL-C ratio to detect MASLD. We also compared the area under the curve (AUC) of the TG/HDL-C ratio, serum TG, and HDL-C. A higher TG/HDL-C ratio was observed among patients with MASLD, compared with patients without MASLD. No differences in the TG/HDL-C ratio were found between participants with MASL and MASH. The greatest AUC was observed for the TG/HDL-C ratio (AUC 0.747, *p* < 0.001) with a cut-off point of 3.7 for detecting MASLD (sensitivity = 70%; specificity = 74.5%). However, no statistically significant differences between the AUC of the TG/HDL-C ratio and TG or HDL-C were observed to detect MASLD. In conclusion, although an elevated TG/HDL-C ratio can be found in patients with MASLD, this marker did not improve the detection of MASLD in our study population, compared with either serum TG or HDL-C.

## 1. Introduction

Metabolic dysfunction-associated steatotic liver disease (MASLD) has become the first cause of chronic liver disease globally [1,2], causing major health, social, and economic burdens; decreasing quality of life; and shortening life expectancy [3,4].

The pathophysiology of MASLD is complex and involves several etiological factors [5,6]. Among them, unhealthy lifestyle habits leading to the development of overweight and obesity are the main contributors to the disease [5,6]. Also, an association between MASLD and additional risk factors related to the metabolic syndrome, such as dyslipidemia, has been demonstrated [7]. Accordingly, elevated circulating levels of triglycerides (TG) and low-density lipoprotein cholesterol (LDL-C), and reduced levels of high-density lipoprotein cholesterol (HDL-C) are often found in patients with MASLD [7]. These lipid abnormalities are associated, in part, to the increased risk of cardiovascular disease observed in MASLD [7,8,9,10].

In recent years, the triglyceride to high-density lipoprotein cholesterol (TG/HDL-C) ratio has been postulated as a useful indirect marker of insulin resistance/type 2 diabetes (T2D) and may be highly predictive for the diagnosis of metabolic syndrome [11,12]. Therefore, Cordero et al. reported that the TG/HDL-C ratio was directly associated with the number of components of metabolic syndrome, and this ratio doubled in patients with metabolic syndrome, compared with healthy subjects [11]. Also, Wang et al. recently showed that a higher TG/HDL-C ratio was a predictor of incidence of T2D [12]. Moreover, a higher TG/HDL-C ratio has been associated with an increased risk for cardiovascular disease in both the general population and subjects at high risk of cardiovascular events [13,14,15].

Previously, some longitudinal and cross-sectional studies have evaluated the clinical utility of the TG/HDL-C ratio as a surrogate marker for MASLD, a condition which is closely related to the metabolic syndrome. In line with this, a higher prevalence of MASLD (assessed by hepatic ultrasonography) has been reported among subjects with a higher TG/HDL-C ratio [16,17,18]. Thus, Wu et al. found that the prevalence of MASLD was lower in the lowest quartile of the TG/HDL-C ratio and observed a higher risk for the disease in participants with more elevated ratios [16]. Similarly, Fan et al. reported a progressive increase in the prevalence of MASLD in parallel with the different quartiles of this ratio [17]. On the other hand, the TG/HDL-C ratio may be able to predict incident MASLD, as observed in some retrospective cohort studies also using ultrasound techniques [19,20]. In this regard, Fukuda et al. showed that the TG/HDL-C ratio may be useful for predicting incident MASLD in healthy Japanese individuals [19], and Chen et al. reported an independent association between this ratio and the incidence of MASLD in a Chinese population without obesity or dyslipidemia [20].

It should be noted, however, that hepatic ultrasonography has several limitations regarding the evaluation of MASLD, including a low sensitivity to detect a liver fat content <20%, and may not be reliable in patients with an elevated body mass index (BMI) (i.e., >40 kg/m^2^) [21]. Therefore, liver biopsy is still considered as the gold standard method for the diagnosis of MASLD, and it is the only technique that permits distinguishing between metabolic dysfunction-associated steatotic liver (MASL) and metabolic dysfunction-associated steatohepatitis (MASH) [21]. Of note, no previous works have considered liver biopsy for the assessment of the utility of the TG/HDL-C ratio for the diagnosis of MASLD.

Hence, in this study we aimed to evaluate the utility of the TG/HDL-C ratio for the identification of MASLD in patients with obesity who had an available liver biopsy.

## 2. Methods

### 2.1. Study Design and Participants

We conducted a case-control study including participants with obesity who underwent metabolic surgery at the Virgen de la Arrixaca University Hospital (Murcia, Spain) from January 2020 to May 2023 and had a concomitant liver biopsy. These participants were enrolled consecutively in this period. Results from this cohort have been previously published, and more details can be found elsewhere [22,23].

Inclusion criteria comprised age between 18 and 65 years old; a BMI ≥ 35 kg/m^2^, or ≥30 kg/m^2^ with significant comorbidities related to obesity; and the availability of liver biopsy. Exclusion criteria included evidence of liver disease different from MASLD; alcohol consumption (>30 g/day for men; >20 g/day for women); and medication that could potentially cause liver steatosis.

Study groups were established according to the histological assessment of liver biopsies, using the steatosis, activity, fibrosis (SAF) classification system as follows: (a) participants without MASLD (normal liver biopsy); (b) participants with MASL (those who presented, at least, grade 1 liver steatosis, with/without ballooning/lobular inflammation, but not both histopathological findings); and (c) participants with MASH (those who presented, at least, grade 1 liver steatosis, with both lobular inflammation and ballooning, with/without liver fibrosis) [24].

This study was approved by the Ethics Research Committee of Virgen de la Arrixaca University Hospital (reference number 2020-2-4-HCUVA) on 31 March 2020, and was conducted according to the principles of the Declaration of Helsinki. Signed informed consent was obtained from all study participants prior to inclusion in this study.

### 2.2. Anthropometric and Biochemical Evaluation

We determined anthropometric measurements, including height, body weight, and waist circumference by standardized methods. BMI was calculated by the formula weight (kg) divided by the square of height (m).

Blood sample collection was performed following an overnight fast of 12 h, and serum was separated by centrifugation. Serum levels of different parameters (glucose, total cholesterol, HDL-C, TG, alanine aminotransferase -ALT-, and aspartate aminotransferase AST-) were determined by standardized methods (Cobas Analyzer c702, Roche). Levels of LDL-C were calculated by Friedewald’s formula (i.e., TC—HDL-C—TG/5). Glycated hemoglobin (HbA1c) levels were determined by the glycohemoglobin analyzer HLC^®^-723G8 (Tosoh Bioscience). Insulin levels were determined in serum using the Cobas Analyzer e801 (Roche). The homeostasis model assessment of insulin resistance (HOMA-IR) was determined by the following formula: insulin (µU/mL) × glucose (mmol/L)/22.5 [25]. The TG/HDL-C ratio was calculated by dividing serum TG by HDL-C.

### 2.3. Liver Biopsies Collection and Processing

The collection and processing of intraoperative liver biopsy samples from patients undergoing metabolic surgery have been described previously [22,23]. Expert liver pathologists from Virgen the la Arrixaca University Hospital and the Experimental Pathology of the Biomedical Research Institute of Murcia evaluated and scored liver biopsies.

### 2.4. Statistical Analysis

Data analysis was performed using the IBM-SPSS statistical software, version 29.0 (IBM Inc., Chicago, IL, USA). The distribution of quantitative variables was evaluated by the Kolmogorov–Smirnov test. Data were expressed as mean ± standard deviation (SD) for variables with a normal distribution, whereas data were expressed as median and interquartile range (Q1–Q3) for variables without a normal distribution.

Comparisons between groups were performed by the ANOVA test for those quantitative variables presenting a normal distribution, and a Kruskal–Wallis test was performed for those quantitative variables without a normal distribution. Proportions were compared by the Pearson’s chi-squared test. The Spearman correlation test was performed to evaluate associations between pairs of biochemical and histopathological variables. MedCalc version 22.009 (MedCalc Software Ltd., Ostend, Belgium) was used for the receiver operating characteristic (ROC) analyses, and the representation of the ROC curves. Significance was set for a *p* value < 0.05.

## 3. Results

### 3.1. Characteristics of the Study Population

In total, 153 participants were included in this study: 53 patients without MASLD, 45 patients with MASL, and 55 patients with MASH. Clinical and anthropometric characteristics of the participants of the study according to liver biopsy can be found in Table 1. In addition, the histopathological findings (i.e., SAF score, liver steatosis, hepatocellular ballooning, lobular inflammation, and liver fibrosis) of subjects in the MASL and MASH groups are shown in Table 2.

Participants with MASLD presented a higher systolic blood pressure, HbA1c, insulin, and HOMA-IR, compared with participants without MASLD. On the other hand, participants with MASH had higher glucose, AST, and ALT levels, compared with participants without MASLD or with MASL. Regarding lipid parameters, higher TG and lower HDL-C levels were found among participants with MASLD, compared with participants without MASLD. A significantly higher TG/HDL-C ratio was also observed in subjects with MASLD. However, no differences in TG, HDL-C, or TG/HDL-C ratio were noted between participants with MASL and MASH.

### 3.2. Correlations between the TG/HDL-C Ratio and Histopathological Parameters in Subjects with MASLD and Obesity

We performed a correlation analysis between the TG/HDL-C ratio, TG and HDL-C levels, and histopathological parameters for the evaluation of MASLD (Table 3). Both TG/HDL-C ratio and serum TG levels showed a positive correlation with liver steatosis, hepatocyte ballooning, lobular inflammation, and SAF score (all *p* values < 0.001), whereas an inverse correlation was found between serum HDL-C levels and these histopathological parameters (all *p* values < 0.001). On the other hand, no significant associations were observed between the TG/HDL-C ratio, serum TG or HDL-C levels, and liver fibrosis.

### 3.3. Accuracy of TG/HDL-C Ratio to Detect MASLD in Patients with Obesity

**Figure 1:**
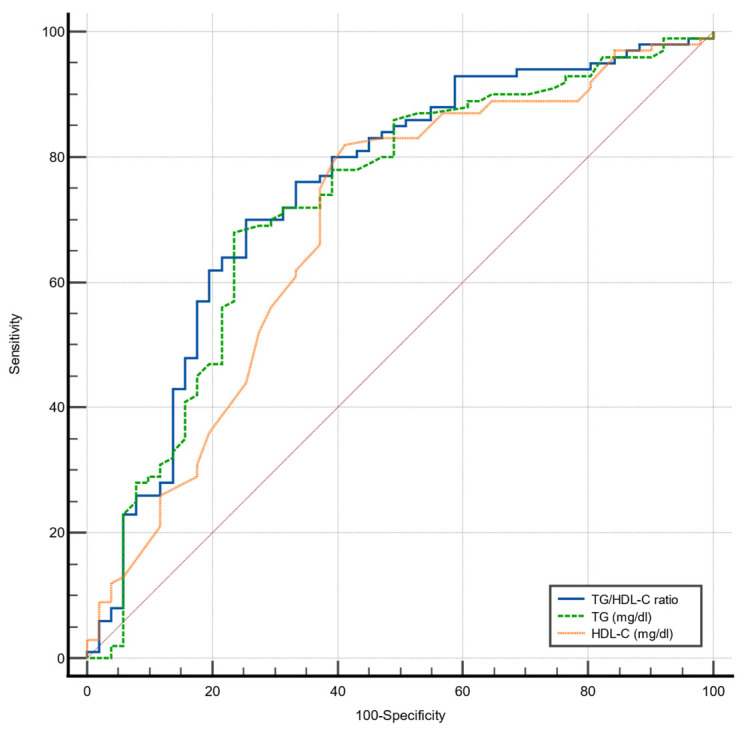
Receiver operating characteristic (ROC) curves of triglycerides (TG); high-density lipoprotein cholesterol (HDL-C) and TG/HDL-C ratio for detecting MASLD in the study population.

In order to evaluate the accuracy of the TG/HDL-C ratio to detect MASLD in our study population, ROC analyses were performed. Also, ROC curves for the diagnostic accuracy of the separated components of this equation (i.e., TG and HDL-C) to detect MASLD were done. Thus, ROC analyses describing the ability of the TG/HDL-C ratio, serum TG levels, and serum HDL-C levels to detect MASLD (including MASL and MASH) in the study population are summarized in Figure 1 and Table 4.

Of note, the greatest AUC was observed for the TG/HDL-C ratio (AUC 0.747, *p* < 0.001) with a cut-off point of 3.7 for detecting MASLD (sensitivity = 70%; specificity = 74.5%). Also, significant AUCs were observed for serum TG (AUC 0.730, *p* < 0.001, with a cut-off value of 161 mg/dl; sensitivity = 68%; specificity = 76.9%), and HDL-C levels (AUC 0.685, *p* < 0.001, with a cut-off value of 46 mg/dl; sensitivity = 82%; specificity = 57.7%). However, no statistically significant differences between the AUC of the TG/HDL-C ratio and either TG or HDL-C were found (*p* = 0.405, and *p* = 0.213, respectively).

We also performed a ROC analysis to evaluate the ability of the TG/HDL-C ratio, serum TG levels, and serum HDL-C levels to detect MASLD specifically in women in the study population (Figure S1 and Table S1). Similar to the analyses performed in the whole study population, significant AUCs were found for the TG/HDL-C ratio, TG, and HDL-C in this subgroup (75.8% of the study population). However, no differences among the TG/HDL-C ratio, TG, and HDL-C levels were found in the AUCs of these parameters in women (*p* = 0.951 and *p* = 0.240 for comparisons between TG/HDL-C ratio and TG levels, and TG/HDL-C ratio and HDL-C levels, respectively).

Finally, although the main aim of this study was to evaluate the utility of the TG/HDL-C ratio to detect biopsy-proven MASLD (as compared with serum levels of TG or HDL-C), we also assessed other non-invasive indexes to predict the disease. Therefore, we considered the hepatic steatosis index (HSI), calculated as [8 × (ALT/AST) + BMI (+2 if T2D, +2 if female)] [26], and the fibrosis-4 index (FIB-4), calculated as [(age × AST)/(platelets × √(ALT)] [27]. We only found a statistically significant p value for the AUC of the FIB-4, but not for the HSI, for predicting MASLD. A greater AUC of the TG/HDL-C ratio was observed as compared with the AUCs of these indexes (Figure S2 and Table S2), finding some trends towards significance for comparisons between the ROC curves.

## 4. Discussion

In this case-control study, we assessed the utility of the TG/HDL-C ratio to diagnose MASLD in patients with obesity, using for the first time liver biopsies as the diagnostic criteria for this condition.

Our main results show that, although an elevated TG/HDL-C ratio was observed in patients with MASLD, this ratio did not improve the detection of MASLD compared with TG or HDL-C in our study population, as shown in the ROC analysis.

Several studies have assessed the utility of the TG/HDL-C ratio for the identification of MASLD, all of them using abdominal ultrasound. Thus, in a large population of Korean adults without diabetes, the TG/HDL-C ratio was demonstrated to be a predictor of MASLD, independently of age, BMI, and waist circumference [28]. Also, Wu et al. reported a direct association between the TG/HDL-C ratio and the degree of hepatic steatosis (i.e., mild, moderate, and severe) [16]. In line with this, we found a positive correlation between TG/HDL-C ratio and liver steatosis assessed by biopsy. Therefore, in light of these findings, it could be suggested that TG/HDL-C ratio may be related to hepatic fat infiltration.

Notably, only a few works have compared the ability of the TG/HDL-C ratio with the separated components of this equation to detect ultrasonographic MASLD. In a cross-sectional study performed in 18,061 healthy subjects, Fan et al. showed that the TG/HDL-C ratio was independently associated with MASLD, and the AUC was significantly higher for the TG/HDL-C ratio than other biochemical parameters, including TG and HDL-C [17]. Similarly, in a population-based cohort study of 4518 healthy subjects followed up over a period of 10 years, Fukuda et al. reported a higher incidence of MASLD in individuals with a higher TG/HDL-C ratio [19]. Interestingly, when all participants with fatty liver (i.e., MASLD and alcoholic fatty liver) were considered, the AUC of the TG/HDL-C ratio was significantly greater than the AUC of TG in men and women, as well as than the AUC of the HDL-C in men [19]. Additionally, in a retrospective cohort study of 9838 Chinese participants without obesity, Chen et al. found a greater AUC of the TG/HDL-C ratio than the AUC of TG or HDL-C for both men and women [20]. Also, among 731 patients with newly diagnosed T2D, the TG/HDL-C was the most accurate marker for the diagnosis of MASLD, compared with other ratios and biochemical parameters, including TG and HDL-C [18]. However, it should be noted that although statistically significant differences between the AUC of the TG/HDL-C ratio and TG or HDL-C were reported, these differences appeared to be clinically less relevant. For instance, in the study by Fan et al., the AUC of the TG/HDL-C ratio was 0.85, and the AUC of TG was 0.84 [17]. On the other hand, the AUC of the TG/HDL-C ratio was 0.67 in men in the study by Fukuda et al., whereas the AUC of TG was 0.66 in this subgroup, also finding very close values for these parameters in women [19]. Accordingly, the large sample sizes included in previous studies may explain, in part, the statistical significance of the results.

Apart from variations in sample size and the diagnostic method of MASLD, additional differences can be found in our study, as compared with previous works. First, whereas most of the aforementioned studies included participants from the general population, our study population mainly included patients with severe obesity (with a mean BMI > 40 kg/m^2^). Second, racial differences might also play a role in the results, as all previous works included Asian participants, whereas this study was performed in Spain and mainly included Caucasian participants. Therefore, these differences may have also contributed to the divergent results found in our study.

As far as we know, this is the first study to assess the relationship between the TG/HDL-C ratio and histopathological parameters related to MASLD. Of note, we found associations between the TG/HDL-C ratio (and also TG and HDL-C) and liver steatosis, hepatocyte ballooning, and lobular inflammation, but not with liver fibrosis. In this regard, in a cross-sectional study performed in 56 participants aged 6–18, with obesity and/or T2D, the highest tertile of the TG/HDL-C ratio was an independent predictor of liver fibrosis, assessed by transient elastography [29]. Even though liver biopsy is the gold standard for the evaluation of fibrosis, given the low proportion of participants with liver fibrosis in our study population, our results should be cautiously interpreted. On the other hand, in a previous cohort of patients undergoing bariatric surgery, serum TG levels were related to advanced stages of biopsy-proven MASLD, including MASH [30]. However, in a recent study conducted in 90 patients with morbid obesity, circulating levels of different particles of the lipid profile (including TG and HDL-C) did not relate to the diagnosis of MASH or histopathological fibrosis [31].

Our results also suggest that the TG/HDL-C ratio might be a useful marker to detect MASLD in patients with morbid obesity compared with other noninvasive markers. In this regard, a greater AUC was found for the TG/HDL-C ratio compared with some of these markers (e.g., HSI), with a trend towards statistical significance for comparisons between the AUCs. However, devoted studies are needed to confirm this hypothesis, also including a larger sample size.

Despite the strengths of our study, it also has some limitations. Firstly, given the relatively small sample size, further large-scale studies based on the histopathological diagnosis of MASLD are needed to evaluate the accuracy of the TG/HDL-C ratio for the identification of the disease. Indeed, larger liver biopsy-based studies might allow for detecting significant differences between the TG/HDL-C ratio and other biochemical parameters in ROC analyses. Secondly, similar to previous studies conducted in patients undergoing bariatric surgery, a predominance of women was observed among the study participants. In this regard, the majority of participants in the study were women, including the large majority of participants without MASLD. This limitation should be taken into consideration, since lipoprotein metabolism (including both HDL-C and TG levels) is different between women and men. Also, we did not consider in our analyses apolipoprotein B-100 levels in the cohort, which might have a relevant role in the detection of MASLD; therefore, this point deserves further investigation. Finally, as previously stated, this study was conducted in Spanish subjects with obesity undergoing bariatric surgery at a single hospital, and these results may not apply to different populations.

## 5. Conclusions

In our study, an elevated TG/HDL-C ratio was found in patients with obesity and biopsy-proven MASLD, but this marker did not improve the detection of MASLD, compared with either serum TG or HDL-C.

## Figures and Tables

**Table 1:** Characteristics of the study population.

	No MASLD (n = 53)	MASL (n = 45)	MASH (n = 55)	*p* Value
Sex (F, M)	50/3	30/15	36/19	0.001
Age (years)	42 (34–51) ^a^	50 (42–55) ^b^	48 (42–55) ^b^	0.007
Weight (kg)	116.6 (108–124)	117.9 (104.8–132.5)	116.4 (97.4–135.6)	0.880
BMI (kg/m^2^)	43.4 ± 5.9	43.6 ± 5.3	43.4 ± 6.9	0.975
WC (cm)	122.4 ± 12.8	126.6 ± 12.8	128.4 ± 15.5	0.088
SBP (mm Hg)	131 (120–143) ^a^	140 (128.5–153.5) ^b^	141 (130–150) ^b^	0.018
DBP (mm Hg)	85 (75–90)	85 (80–93)	82.5 (76.8–90.2)	0.563
Glucose (mg/dL)	88 (80–96.5) ^a^	94 (85.5–104) ^b^	101 (89–123) ^c^	<0.001
HbA1c (%)	5.4 (5.1–5.8) ^a^	5.7 (5.4–6.1) ^b^	5.8 (5.6–6.8) ^b^	<0.001
Insulin (μIU/mL)	8.6 (5.8–15.2) ^a^	13.1 (9.3–18.5) ^b^	14.7 (9.2–26.7) ^b^	0.005
HOMA-IR	1.9 (1.2–3) ^a^	3.1 (2.2–4.3) ^b^	3.7 (2.1–7.8) ^b^	<0.001
Cholesterol (mg/dL)	159 ± 30.6	165.1 ± 32	163.4 ± 31.6	0.609
HDL-C (mg/dL)	48 (37.3–54) ^a^	39 (33–45) ^b^	38 (33–44) ^b^	0.001
LDL-C (mg/dL)	87.6 ± 26.8	85.7 ± 27.9	85.4 ± 30.8	0.920
Triglycerides (mg/dL)	124 (103.8–161) ^a^	175 (148.5–229.5) ^b^	184 (153–230) ^b^	<0.001
TG/HDL-C ratio	2.9 (2.2–3.9) ^a^	4.3 (3.4–7.4) ^b^	4.6 (3.3–6.6) ^b^	<0.001
AST (U/L)	16 (13–19.3) ^a^	18 (14.3–21.5) ^a^	21.5 (17.8–28.8) ^b^	<0.001
ALT (U/L)	14.5 (11–19) ^a^	18 (14–28.5) ^b^	24 (19–40) ^c^	<0.001

Values are presented as mean ± standard deviation (SD) for normally distributed continuous variables, and as median (interquartile range, Q1–Q3) for those continuous variables without a normal distribution. *p* values were determined by the performance of an ANOVA test in normally distributed continuous parameters, and the Kruskal–Wallis test was performed to compare continuous parameters without a normal distribution, followed by a Bonferroni post hoc analysis. Proportions were compared using Pearson’s chi-squared test. ALT, alanine aminotransferase; AST, aspartate aminotransferase; BMI, body mass index; DBP, diastolic blood pressure; F, female; HbA1c, glycated hemoglobin; HDL-C, high-density lipoprotein cholesterol; HOMA-IR, homeostatic model assessment of insulin resistance; LDL-C, low-density lipoprotein cholesterol; M, male; MASH, metabolic dysfunction-associated steatohepatitis; MASL, metabolic dysfunction-associated steatotic liver; MASLD, metabolic dysfunction-associated steatotic liver disease; SBP, systolic blood pressure; TG, triglycerides; WC, waist circumference. Statistically significant differences between the correspondent groups are shown by different superscript letters. Statistical significance was set for a *p* value < 0.05.

**Table 2:** Histopathological findings of subjects with MASL and MASH.

	MASL (n = 45)	MASH (n = 55)	*p* Value
SAF score (0/1/2/3/4/5/6/7/8/9)	(0/20/17/7/1/0/0/0/0)	(0/0/0/19/13/13/6/2/1/1)	˂0.001
Steatosis (0/1/2/3)	(0/34/11/0)	(0/30/14/11)	0.009
Hepatocellular ballooning (0/1/2)	(26/18/1)	(0/43/12)	˂0.001
Lobular inflammation (0/1/2/3)	(41/4/0/0)	(0/41/14/0)	˂0.001
Fibrosis (0/1/2/3)	(44/1/0/0)	(42/12/1/0)	0.009

Values are presented as frequencies. *p* values were calculated using a Pearson’s chi-squared test, considering a *p* value < 0.05 as significant. SAF, steatosis, activity, and fibrosis; MASL, metabolic dysfunction-associated steatotic liver; MASH, metabolic dysfunction-associated steatohepatitis.

**Table 3:** Correlations between serum TG, HDL and TG/HDL-C ratio, and histopathological parameters related to MASLD.

	TG	HDL-C	TG/HDL-C
SAF score	0.366 **	−0.314 **	0.383 **
Steatosis	0.393 **	−0.333 **	0.422 **
Hepatocyte ballooning	0.305 **	−0.228 **	0.291 **
Lobular inflammation	0.247 **	−0.232 **	0.261 **
Fibrosis	0.099	−0.136	0.111

** Indicates *p* value < 0.001. HDL-C, high-density lipoprotein cholesterol; MASLD, metabolic dysfunction-associated steatotic liver disease; SAF, steatosis, activity, and fibrosis; TG, triglycerides; TG/HDL-C, triglyceride to high-density lipoprotein cholesterol ratio.

**Table 4:** Receiver operating characteristic (ROC) analyses describing the ability of TG/HDL-C ratio, TG, and HDL-C to detect MASLD in the study population (no MASLD, n = 53; MASLD, n = 100).

	AUC	95% CI	Cut-Off Value	Sensitivity	Specificity	*p* Value
TG/HDL-C ratio	0.747	0.670–0.814	3.7	70.0%	74.5%	<0.001
TG	0.730	0.653–0.799	161	68.0%	76.9%	<0.001
HDL-C	0.685	0.605–0.758	46	82.0%	57.7%	<0.001

AUC, area under the curve; CI, confidence interval; HDL-C, high-density lipoprotein cholesterol; MASLD, metabolic dysfunction-associated steatotic liver disease; TG, triglycerides; TG/HDL-C, triglyceride to high-density lipoprotein ratio. Binomial exact confidence intervals for the AUC are given.

## Data Availability

The data presented in this study are available on reasonable request to the corresponding authors.

## Supplementary Materials

The following supporting information can be downloaded at: https://www.mdpi.com/article/10.3390/nu16091310/s1, Supplementary Figure S1. Receiver operating characteristic (ROC) curves of triglycerides (TG); high-density lipoprotein cholesterol (HDL-C) and TG/HDL-C ratio for detecting MASLD in women of the study population. Supplementary Figure S2. Receiver operating characteristic (ROC) curves of the triglyceride to high-density lipoprotein cholesterol (TG/HDL-C) ratio, hepatic steatosis index (HSI) and fibrosis-4 index (FIB-4) for detecting MASLD in the study population. Supplementary Table S1. Receiver operating characteristic (ROC) analyses describing the ability of TG/HDL-C ratio, TG, and HDL-C to detect MASLD in women (no MASLD, n = 50; MASLD, n = 66). Supplementary Table S2. Receiver operating characteristic (ROC) analyses describing the ability of the TG/HDL-C ratio, HSI, and FIB-4 to detect MASLD in the study population.
