# Supplementary material for: Triglyceride to HDL Cholesterol Ratio for the Identification of MASLD in Obesity: A Liver Biopsy-Based Case-Control Study
Source: Nutrients. 2024 Apr 27;16(9):1310. doi: 10.3390/nu16091310 (PMC11085202; doi:10.3390/nu16091310)

Supplementary Figure S2. Receiver operating characteristic (ROC) curves of the triglyceride to high-density lipoprotein cholesterol (TG/HDL-C) ratio, hepatic steatosis index (HSI) and fibrosis-4 index (FIB-4) for detecting MASLD in the study population.

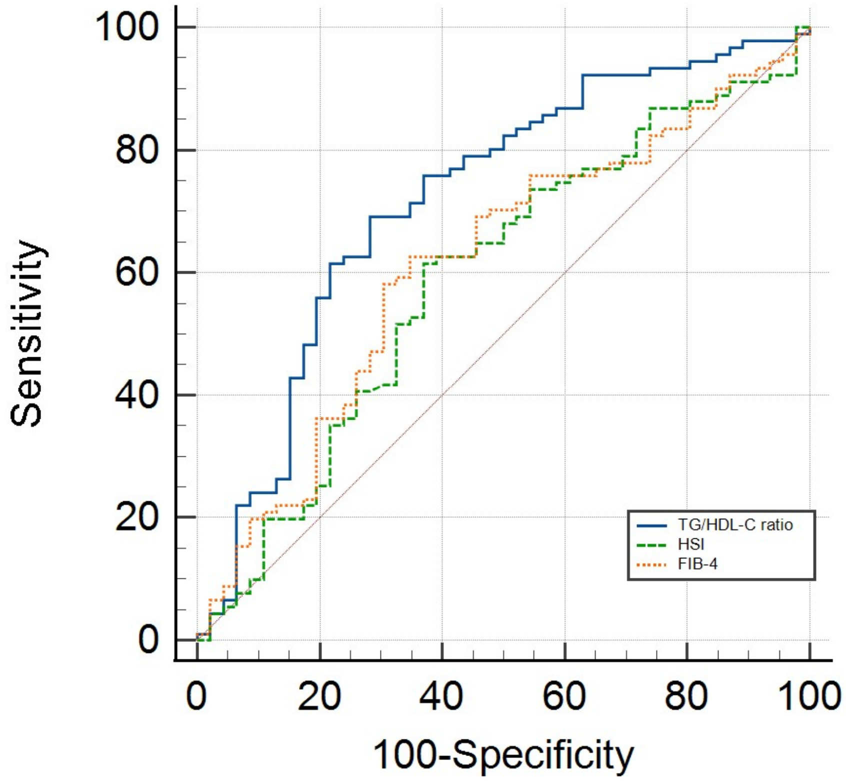

Supplement: Supplementary file 1 [file nutrients-16-01310-s001.zip › Supplementary Figure S2.pdf]
