# Supplementary material for: Triglyceride to HDL Cholesterol Ratio for the Identification of MASLD in Obesity: A Liver Biopsy-Based Case-Control Study
Source: Nutrients. 2024 Apr 27;16(9):1310. doi: 10.3390/nu16091310 (PMC11085202; doi:10.3390/nu16091310)
Supplement: Supplementary file 1 [file nutrients-16-01310-s001.zip › Supplementary Table S1.pdf]

Supplementary Table S1. Receiver operating characteristic (ROC) analyses describing the ability of TG/HDL-C ratio, TG, and HDL-C to detect MASLD in women (no MASLD, n=50; MASLD, n=66)

	AUC	95% CI	Cut-off value	Sensitivity	Specificity	P value
TG/HDL-C ratio	0.700	0.607 – 0.782	3.7	60.6%	75%	<0.001
Triglycerides	0.704	0.611 – 0.785	161	63.6%	75.5%	<0.001
HDL-C	0.631	0.536 – 0.719	46	74.2 %	61.2%	0.0179

MASLD, metabolic dysfunction-associated steatotic liver disease; AUC, area under the curve; CI, confidence interval; TG, triglycerides; HDL-C, high-density lipoprotein cholesterol.
