# Supplementary material for: Triglyceride to HDL Cholesterol Ratio for the Identification of MASLD in Obesity: A Liver Biopsy-Based Case-Control Study
Source: Nutrients. 2024 Apr 27;16(9):1310. doi: 10.3390/nu16091310 (PMC11085202; doi:10.3390/nu16091310)
Supplement: Supplementary file 1 [file nutrients-16-01310-s001.zip › Supplementary Table S2.pdf]

Supplementary Table S2. Receiver operating characteristic (ROC) analyses describing the ability of the TG/HDL-C ratio, HSI, and FIB-4 to detect MASLD in the study population.

	AUC	95% CI	Cut-off value	Sensitivity	Specificity	P value	P value for comparisons*
TG/HDL-C ratio	0.747	0.670 – 0.814	3.7	70.0%	74.5%	<0.001	
HSI	0.592	0.506 – 0.675	52.6	62.4%	61.7%	0.074	0.069
FIB-4	0.615	0.530 – 0.696	0.74	62.0%	65.3%	0.020	0.143

AUC, area under the curve; CI, confidence; FIB-4, fibrosis-4 index; HSI, hepatic steatosis index MASLD, metabolic dysfunction-associated steatotic liver disease; TG/HDL-C, triglyceride to high-density lipoprotein cholesterol ratio. Binomial exact confidence intervals for the AUC are given. *P value for comparisons with TG/HDL-C ratio (only participants with all data available, n= 137).
